# Delayed Presentation of Renocolic Fistula at 4 Months after Blunt Abdominal Trauma

**DOI:** 10.1155/2011/103497

**Published:** 2011-02-22

**Authors:** Sang Don Lee, Tae Nam Kim, Hong Koo Ha

**Affiliations:** Department of Urology, Pusan National University School of Medicine, 305, Gudeok-Ro, Seo-Ku, Busan 602739, Republic of Korea

## Abstract

Causes of previously reported reno-colic fistulas included primary renal and colonic pathologic states involving infectious, malignant or other inflammatory processes. However, reno-colic fistula after renal injury is extremely uncommon. We report an unusual delayed presentation of reno-colic fistula that occurred at 4 months later after blunt abdominal trauma.

## 1. Introduction

There were several reports of renocolic fistula after penetrating trauma, renal stone and iatrogenic renal traumas and renal diseases. However, renocolic fistula after blunt abdominal trauma is extremely uncommon, and this report is the first case report of delayed presented renocolic fistula after blunt abdominal trauma.

## 2. Case Presentation

**Figure 1:**
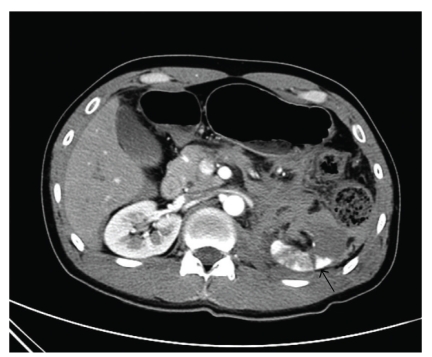
Computed tomography after abdominal trauma reveals left renal injury (grade IV, black arrow) and perirenal hematoma (white arrow).

A 36-year-old male visited of our facility complaining air bubbles during voiding. He had a past history of conservative management in another hospital for 1 month due to in-car traffic accident 4 months ago. On the radiologic examination in the previous hospital, there was only left renal injury (grade IV/V) and perirenal hematoma with no evidence of renocolic fistula (Figure 1) and mild hemothorax. 

The physical examination and laboratory findings were not specific for the renocolic fistula. He had no intestinal or urinary disturbances except air bubbles in the urine over several days.

**Figure 2:**
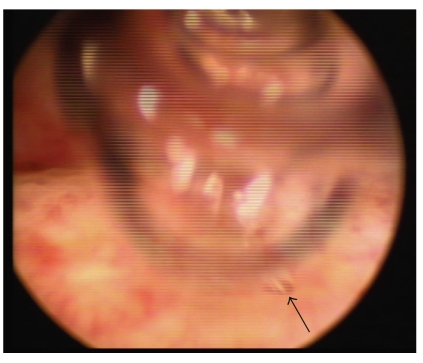
Cystoscopy demonstrates air bubbles jetting at the left ureteral orifice (arrow).

**Figure 3:**
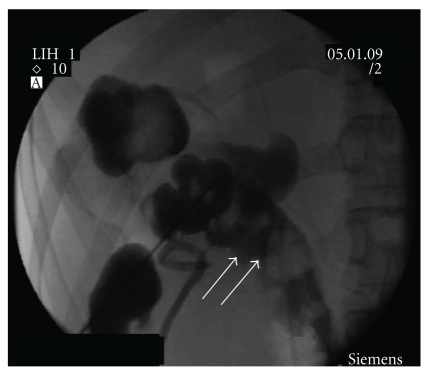
Antegrade pyelogram shows the fistula from the left renal pelvis into the proximal descending colon. Contrast media is flowing backward into the descending colon (arrow).

**Figure 4:**
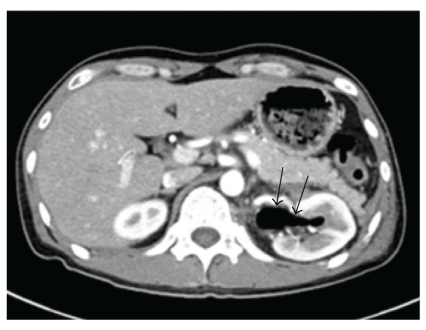
CT of the abdomen and pelvis. There is a gas shadow in the left renal pelvis (black arrows).

On the cystoscopy, we found a diffuse mucosal edema suggesting cystitis and an air bubble jetting in the left ureteral orifice, but fecal materials were not seen in the bladder and around the left ureteral orifice (Figure 2). On the radiologic examination, an accumulation of contrast medium was seen in the left colon on intravenous pyelography. On the antegrade pyelography through percutaneous nephrostomy, the left ureter showed a normal feature but there was contrast congestion in the splenic flexure of the colon (Figure 3). Computed tomography (CT) of abdomen and pelvis revealed multiple gas shadows in the left renal pelvis and the bladder associated with a fistula tract between the lower area of the left pelvis and the splenic flexure of the colon with inflammatory changes (Figure 4) The right kidney showed normal contrast enhancement without any abnormality. 

With a diagnosis of renocolic fistula, the patient was subsequently explored through a left 11th transcostal flank incision with transperitoneal approach. The splenic flexure and the descending colon were tightly adherent to the kidney around the fistula. The fistula was identified between the left renal pelvis and splenic flexure of the colon. Partial colectomy was performed. There was no evidence of foreign body involving in the formation of the fistula. The lower part of the kidney revealed the necrotic change, and the renal parenchyma was replaced by the yellowish inflammatory tissues. Because of severe adhesion and scarring around the renal hilum and the renal pelvis, left nephrectomy was performed.

 There were severe inflammatory changes around the resected colon and the kidney on the pathologic examination. The patient is presently doing well for 12 months without any complications after the operation and shows normal renal function.

## 3. Discussion

A review of the authors' literature found approximately one hundred case reports of renoalimentary fistula. Renocolic fistulas were most common, accounting for about 60% of fistulas reported by Bissada et al. [1]. In his review, other fistula locations included fistulous tracts to the esophagus, stomach, duodenum, small bowel, appendix, and rectum. In most cases, renocolic fistulas originated from renal diseases [2]. However, renocolic fistula associated with blunt renal trauma has been extremely rarely reported and this is the first report of delayed presentation of renocolic fistula that occurred at 4 months later.

Trauma could be identified as a cause of reno-alimentary fistula in the previously reported cases. Iatrogenic injuries, both surgical and percutaneous procedures to the renal system, also may cause renoalimentary fistula [3]. One case of a gunshot wound causing inflammatory changes resulting in a renoduodenal fistula has been reported. In PubMed searching, we could not find any report using “blunt trauma”, “reno”, “pyelo”, “alimentary”, “colic” and “fistula”. And fistula in this case was not found at the time of renal trauma and there was no specific symptoms and signs for 4 months. Delayed presented fistula after blunt trauma is extremely rare and we did not find any one similar to this case following previous blunt trauma.

The diagnosis of the fistula is made by radiographic evaluations. Historically, retrograde pyelography has been the radiologic procedure of choice to identify a fistula. Rodney et al. reported that only 14% and 15% of the fistulas were identified by intravenous urography and upper gastrointestinal studies, respectively [4]. However, because retrograde pyelography revealed the fistula in 64% of the patients, Desmond et al. suggested retrograde pyelography was ideal diagnostic modality [5]. However, methods diagnosing the fistula have subsequently changed. We suspected the reno-alimentary fistula through air bubbles at the cystoscopy and confirmed the fistula tract on CT scan. 

The mechanism of the fistula in the current case is unclear, but there may be two possibilities. First, the descending colon injury had accompanied at blunt abdominal trauma, and this caused the inflammation around the kidney and induced local abscess, which corroded into the renal hematoma and made the fistula with the collection system of kidney. Second, the renal pelvis might have leaked around the perirenal space. Then, there could be a formation of local abscess in the lower pole of kidney, which causes subsequent fistula with the colon. However, we could not find any sign of injury in alimentary tract and leakage of contrast from kidney on CT that was performed in previous hospital.

Treatment options for a reno-alimentary fistula are varied and should be individualized according to the patient circumstances. Some authors reporting cases of early diagnosed iatrogenic perforations and fistulas suggest that nonsurgical treatment including antibiotics and bowel rest is adequate therapy [6]. Herbert et al. reported conservative treatment of renocolic fistula including ureteral stent, drain and percutaneous nephrostomy was successful [2]. But Ginsberg et al. reported conservative treatment in reno-duodenal patient was unsuccessful [7]. Traditionally, the management of renocolic fistula consists of nephrectomy, excision of the fistulous tract, and resection of the involved bowel with primary anastomosis [8]. In this case, nephrectomy and partial resection of colon was performed because of the associated severe fibrosis, scarring and inflammatory changes around the renal hilum and the renal pelvis, and the possibilities of long-term complications including persistent infections, sinuses and sepsis after renal sparing management.

 Recently, most authors have favored primary repair of all penetrating colon wounds [8]. Others have selected several criteria for primary repair versus staged repair, and suggest that such criteria as the presence of other organ system injuries should preclude primary repair [9]. George et al. reported 95 patients who had primary repair of colon injuries; about 30% of patients had associated renal injuries [10]. As illustrated in this case, associated injuries to the renal parenchyma should be considered as a risk factor for primary colon repair [10]. Rodney et al. also suggested nephrectomy and primary repair of alimentary tract was considered the treatment of choice in patient with reno-alimentary fistula because there were long-term complications including persistent infections, sinuses and sepsis in patient with renal sparing management [4].
